# Focal Point Evaluation of Energies for Tautomers and Isomers for 3-hydroxy-2-butenamide: Evaluation of Competing Internal Hydrogen Bonds of Types -OH…O=, -OH…N, -NH…O=, and CH…X (X=O and N)

**DOI:** 10.3390/molecules26092623

**Published:** 2021-04-30

**Authors:** Zikri Altun, Erdi Ata Bleda, Carl Trindle

**Affiliations:** 1 Physics Department, Marmara University, Göztepe Kampus, Istanbul 34772, Turkey; zikalt@marmara.edu.tr (Z.A.); ata.bleda@marmara.edu.tr (E.A.B.); 2 Chemistry Department, University of Virginia, Charlottesville, VA 22902, USA

**Keywords:** intramolecular hydrogen bonding, high-accuracy extrapolation methods, QTAIM, non-covalent interactions, local vibrational modes

## Abstract

The title compound is a small molecule with many structural variations; it can illustrate a variety of internal hydrogen bonds, among other noncovalent interactions. Here we examine structures displaying hydrogen bonding between carbonyl oxygen and hydroxyl H; between carbonyl oxygen and amino H; hydroxyl H and amino N; hydroxyl O and amino H. We also consider H-bonding in its tautomer 2-oxopropanamide. By extrapolation algorithms applied to Hartree-Fock and correlation energies as estimated in HF, MP2, and CCSD calculations using the cc-pVNZ correlation-consistent basis sets (N = 2, 3, and 4) we obtain reliable relative energies of the isomeric forms. Assuming that such energy differences may be attributed to the presence of the various types of hydrogen bonding, we attempt to infer relative strengths of types of H-bonding. The Atoms in Molecules theory of Bader and the Local Vibrational Modes analysis of Cremer and Kraka are applied to this task. Hydrogen bonds are ranked by relative strength as measured by local stretching force constants, with the stronger =O…HO- > NH…O= > -OH…N well separated from a cluster > NH…O= ≈ >NH…OH ≈ CH…O= of comparable and intermediate strength. Weaker but still significant interactions are of type CH…N which is stronger than CH…OH.

## 1. Introduction

The concept of hydrogen bonding has evolved considerably over its century of history [1,2]. The anomalous properties of water prompted the first suspicion of an attractive interaction between a hydrogen atom in one water molecule with the oxygen of another (HOH…OH_2_). Similar phenomena involving N and F were soon recognized as well. H bonding has been recognized as a primarily electrostatic phenomenon expressed by its effect on vibrational frequencies (most often red-shifting the OH stretch), molecular structure (short X…HY distances, where X and Y are electronegative atoms F, O, N etc.), and characteristic NMR parameters (owing to charge shifts) to mention the most prominent. The concept of H bonding has been expanded from its original context so to include a number of surprising interactions, involving atoms other than oxygen, fluorine, and nitrogen [3,4]; “strong” H-bonding [5]; and “anomalous” (blue-shifting) H bonding [6]. Further expansion of the concept also has been recognized [7].

The computational modeling of hydrogen bonding was reviewed in 1997 [8] and 1999 [9], in 2006 [10], and in 2009 [11]. The definition of hydrogen bonding has been formalized by IUPAC [12]. IUPAC’s criteria for recognition of H bonding in a structure X-H…YZ include: origins of bonding (largely electrostatic, but also including contributions from charge transfer and dispersion); polarization of the XH bond; geometry of the structure (XH…Y near linearity); distortions from reference structures (extension of the XH bond, with impact on vibrational frequencies); effects on NMR spectra including deshielding of H and coupling of X to Y. In the language of the Atoms in Molecules theory [13], a hydrogen bond is associated with presence of a bond path H…Y including a bond critical point (BCP) between H and Y. We discuss this criterion below.

Recent collections of studies of intramolecular H bonding include the Molecules special issue from 2017 edited by S. Scheiner [14] and a follow-up 2019 special issue of Molecules edited by G. Sanchez [15]. The current special issue gives primary attention to computational studies. Interest in evaluating the strength of H bonds continues [16,17] and is an important part of this special issue of Molecules.

Intramolecular hydrogen bonding has a significant effect specifically on conformational preference in systems of importance in biochemistry, as various types of H bonding can occur in various conformations and isomers. The intramolecular H bonds in histidine have been described by Yannacome, Sethlo, and Kraka (YSK) [18]. To enhance insight into the relative strength of H bonds in context these authors brought together descriptors from Bader’s theory of atoms in molecules (AIM) and the reduced density gradient from analysis of noncovalent interactions (NCI), both of which are described below. According to YSK two local measures of bond strength, the density at a BCP and the local stretching force constant are related by a power law. An analogous l link between a local stretching force constant (and hence that density) and bond energy seems eminently reasonable [19,20]. One particularly simple connection, between bond strength and the potential energy density at a Bond Critical Point, has been proposed [21]. However there is a conceptual issue in relating a strictly local property (such as the density at a specific point) to what is not a strictly local property, the energy associated with molecular rearrangement, especially dissociation.

The IUPAC AIM bond path and critical point criteria [12] for the existence of a hydrogen bond is subject to interpretation. Sometimes a path and its critical point seem much at variance with intuitive notions of bonding [22,23,24,25,26]. Bader recognized this awkward conflict, [27] and a proposal that these topological entities be called “line” CPs has been offered. [28] The issue is particularly troubled for weak interactions, which vary in directionality depending on the extent of electrostatic character [29].

In this work we study acetoacetamide and variants of its tautomer 1-amino-1-hydroxyacetone. Bauer and Wilcox addressed the issue of relative stability of the similar but simpler systems malonaldehyde and acetyl acetone [30], concluding that the enol form was the more stable. Intramolecular hydrogen bonding in many hydroxycarbonyl systems has been studied by Afonin and Vashchenko [31]. All the systems they reported have =O…HO- interactions. The keto-enol isomerization of many substituted diketones has been studied by Belova et al. [32] In the unsubstituted system propane-1,3-dione which we can call (H, H), the enol form was favored in internal energy by 14.6 kJ/mol; ΔG = 6.2 kJ/mol according to the CBS-4 thermochemical scheme [33]. That scheme favors the enol of the (CH_3_, CH_3_) species acetylacetone by a Gibbs energy of 11.0 kJ/mol [26]. MP2 and B3LYP calculations by Belova et al. [32] favor the enol form by ca. 10 kJ/mol. Their systems include (R1, R2) = (NH_2_, CH_3_) of central interest here. The DFT model B3LYP/aug-cc-pVTZ placed a keto form above an enol form by 13 kJ/mol; the O…O distance in the enol was found to be 2.55 Å and the O…H distance was 1.638 Å in the enol. The enol form was evidently the structure we label TWO; see below. The authors addressed the issue of H-bond strength in their systems, recognizing the long-established link between H bonding and the OH stretching frequency shift to the red relative to the value for a OH group not involved in H bonding. Strong correlations between O…O shortening, O…H shortening and OH lengthening are noted.

## 2. Results

In the following sections we define the systems under study, describe the extrapolation techniques by which we obtain accurate relative energies, and lay out results of AIM analysis of their charge distributions. Complementary information on non-covalent interactions is provided by graphical representation of the reduced density gradient. We interpret the vibrational spectra for all species, and recover the force constants for significant local modes by the LMODEA algorithms. Those force constants are found to be useful indices of relative intramolecular bond strength. All these analyses are described in detail in the section on methods and software.

### 2.1. Systems under Study

In the following enumeration of structures under study, actual reaction paths are not described; there is instead a series of logical connections between related species.

**Figure 1:**
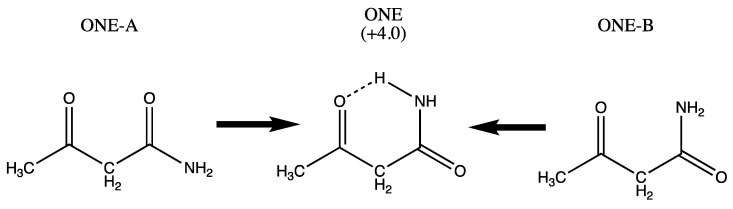
Diketone variant ONE with NH…O hydrogen bonding. Species ONE-A and ONE-B (which lacks the NH…O = H bond) proceed spontaneously upon optimization to ONE, which lies about 4.0 kJ/mol above the most stable isomer TWO.

We begin with the tautomer ONE (acetoacetamide) which has a saturated link and two carbonyl groups. Species ONE-A and ONE-B obtained by rotation of the amido and acyl groups revert spontaneously to species ONE as shown in Figure 1, and are not assigned a relative energy.

**Figure 2:**
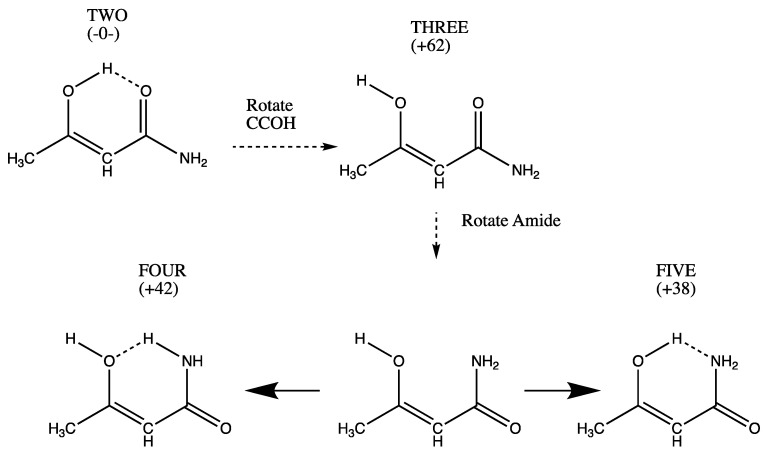
Enolic form THREE can assume forms FOUR with NH...O and FIVE with OH...N H-bonding.

**Figure 3:**
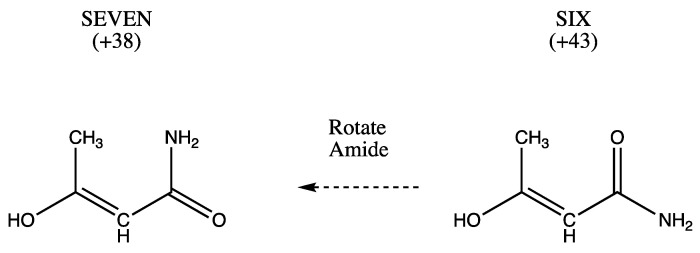
SIX, which may exhibit CH...O= hydrogen bonding, can form SEVEN by a rotation of the amide group. SEVEN may contain CH...N hydrogen bonding.

**Figure 4:**
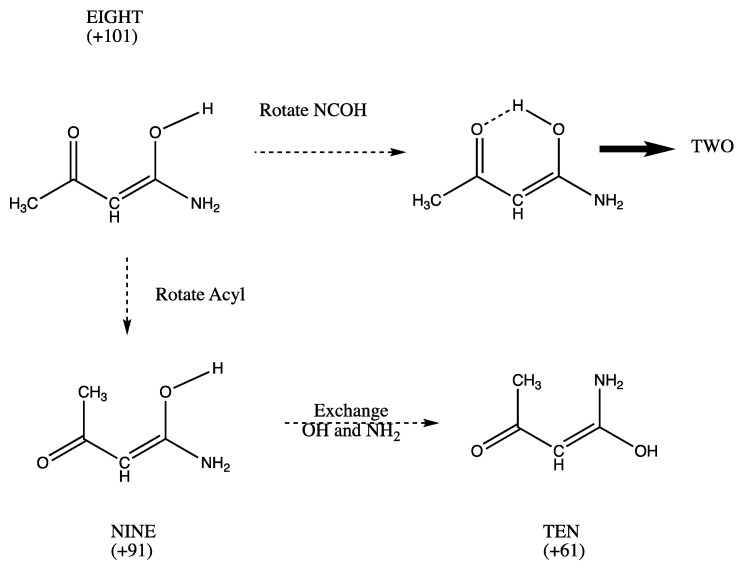
A high energy isomer EIGHT with no hydrogen bonding can achieve OH…O = hydrogen bonding; the resultant species rearranges spontaneously to stable TWO. By rotation of the acyl fragment EIGHT can produce NINE which may have CH…OH hydrogen bonding. Swapping OH and NH_2_ produces TEN which may contain a CH…NH_2_ interaction.

**Figure 5:**
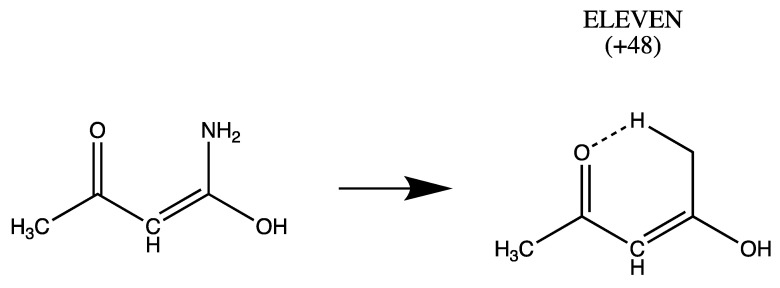
Swapping amino and hydroxyl groups in EIGHT produces a form (unlabeled) which spontaneously forms ELEVEN which has NH…O= hydrogen bonding.

All other systems TWO to ELEVEN (shown in Figure 2, Figure 3, Figure 4 and Figure 5) have an unsaturated link and a hydroxyl group at either the methyl-substituted C (CH_3_-C-OH) or at the amino-substituted C group (NH_2_-C-OH). The most stable of all species, TWO, has an evident OH…O = hydrogen bond. Rotating the CH_3_-C-O-H dihedral forms THREE, which lacks that hydrogen bond (Figure 2). Simple torsion of THREE around the CC-amide bond produces an unstable form (unlabeled) which upon optimization can establish either a NH donor -OH acceptor H bond (FOUR) or an N acceptor OH-donor H bond (FIVE).

Beginning with THREE, exchanging methyl and hydroxyl produces SIX. A subsequent rotation of the amide produces SEVEN (Figure 3).

We turn to structures with an unsaturated link and one carbonyl group at the methyl-substituted C. The system EIGHT which lacks H bonding occupies a high-energy relative minimum (Figure 4). If the OH group is positioned to donate a H bond to the carbonyl oxygen, the system spontaneously rearranged to species TWO. Rotating the acyl group produces species NINE. Exchange of amino and hydroxyl groups in NINE produces TEN. NINE and TEN may be stabilized by CH…OH and CH…N interactions.

On the other hand, exchange of amino and hydroxyl groups in EIGHT produces a species lacking H bonding (unlabeled). Upon optimization it rearranges spontaneously to ELEVEN which is stabilized by > NH…O= H bonding (Figure 5).

### 2.2. Extrapolation of Accurate Relative Energies

The numerical values (kJ/mol) in Figure 1, Figure 2, Figure 3, Figure 4 and Figure 5 are the result of the extrapolations described in the methods section. Estimates of electronic energies from the G4 thermochemical scheme agree within 1–2 kJ/mol with our extrapolated values. Detailed tables of energies obtained with RHF, MP2, and CCSD Hamiltonians in Dunning basis sets cc-pVNZ with N = 2, 3, and 4 are provided in Supplemental Information. A broad overview is set forth in Table 1.

There is a rough clustering of H bonding types. Species ONE and TWO with NH…O= and OH…O= hydrogen bonding are most stable, while systems THREE and EIGHT with little or no H bonding are relatively unstable. However systems with presumably much weaker CH…O or CH…N interactions (SIX, SEVEN, and TEN) are not entirely separated from systems incorporating XH…Y (X, Y = O and N) which are generally thought to be stronger.

It appears that the relative stability, a global property for each of the molecules in question, is not simply explained as a consequence only of differences in local H bonding. Our task, to describe the hydrogen bonds, needs more local analysis.

### 2.3. Atoms in Molecules (AIM) Characterization of Interactions

The Quantum Theory of Atoms-in-Molecules, which defines local properties of the charge distribution at significant points, has been widely employed to characterize bonding of many kinds. (See further discussion in the Methods and Software section below.) Table 2 collects the electron density and its Laplacian at bond critical points, with the associated kinetic energy density G and the potential energy V. The total charge *Q(H)* in the basin containing H atom and the delocalization index *δ(H, B)* are shown as well. The delocalization index is a measure of the number of electrons shared between two basins, and is related to the extent of covalent bonding and, indirectly, to bond strength. Kraka and co-workers [34] define an empirical bond order *n* derived from the density at a BCP.

*n* = 0.54 *ρ*^0.32^ (1)

The positive values of the Laplacian (Table 2) indicate that the interaction is between closed shells. The values of bond order indicate that the strongest H bond is the link OH…O of TWO at 0.221, while the bond orders descend from 0.191 to 0.180 to 0.164 to 0.158 for ELEVEN, FIVE, ONE, and FOUR. These all have O to N hydrogen bonds. CH…X interactions are found in SIX (0.155), TEN (0.142), SEVEN (0.138), and NINE (0.129). OH seems to be a weaker H-bond acceptor than carbonyl =O. We do not assign a bond order to THREE and EIGHT, despite the presence of appreciable density at BCPs between O atoms. See further discussion on THREE and EIGHT below and in the section on non-covalent interactions.

An energy density diagnostic adopted by Cremer and Kraka [34] identifies interactions as mainly electrostatic (if H = G + V > 0) or mainly covalent (if H < 0). [35,36,37,38,39,40] By this criterion the only covalent interactions are for TWO (-OH…O=), FIVE (-OH…NH) and ELEVEN (=O…HN). These also have the shortest X…Y distances (2.538, 2.631, and 2.617 Å). ONE, which has an =O…HN- interaction has crossed over to be predominantly electrostatic. This may be attributed to its greater O…N distance, 2.789 Å, since the electrostatic interaction is of longer range than the covalent interaction which depends on orbital overlap.

H > O for the systems with no plausible H bonding (THREE and EIGHT) and those for which CH…X hydrogen bonding is conceivable (SIX, SEVEN, NINE, and TEN). It is notable that ONE and FOUR with =O…HN and HO…HN interactions are to be considered electrostatic according to the H diagnostic.

**Figure 6:**
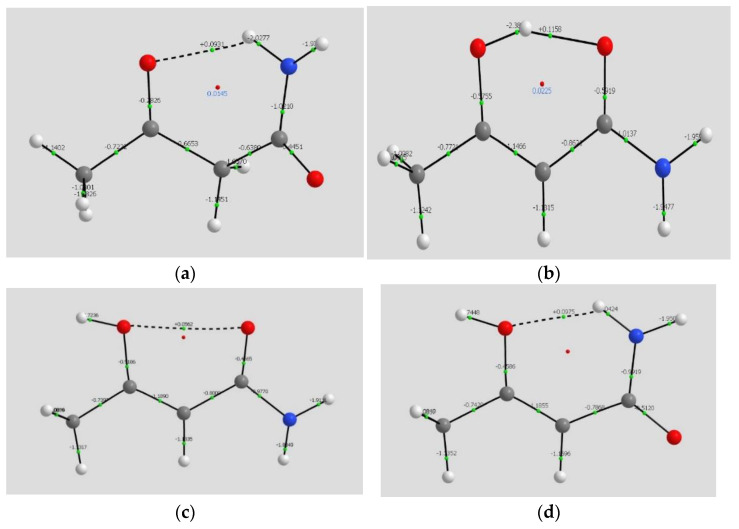
Atoms in Molecules analysis of ωB97XD/cc-pVTZ density. Ring CPs in red, BCPs in green. Laplacian values at BCPs are shown. (**a**) Species ONE, with NH…O= hydrogen bonding; (**b**) Species TWO, with OH…O= hydrogen bonding; (**c**) Species THREE with repulsive noncovalent interaction; (**d**) Species FOUR, with NH…OH.

Several images representative of interactions as characterized by AIM appear in Figure 6 The complete set is to be found in Supplemental Information.

The diagrams display the “bond paths” (solid and dashed lines) and the bond (or line) critical points (green) for each species, and the ring critical points as well (red). The paths and BCPs close a ring; the location of the ring critical point is related to the strength of the ring closure. In the weaker ring closing interactions, the RCP approaches the BCP, as in THREE and EIGHT. For CH…X interactions, the RCP is further removed from the BCP, and for stronger hydrogen bonds the separation is even greater.

Our emphasis has been on the BCPs associated with hydrogen bonding and the properties of density at those points. What are we to make of THREE and EIGHT, with O…O paths and a substantial positive Laplacian considerable density at the (path) critical point? In common with the other systems, the Laplacians show that the interaction depletes density at the CP, as is characteristic of interactions between closed shells. To deal with such cases we turn to the reduced density gradient, which diagnoses non-covalent interactions.

### 2.4. Non-Covalent Interaction (NCI) Characterization of Interactions

**Figure 7:**
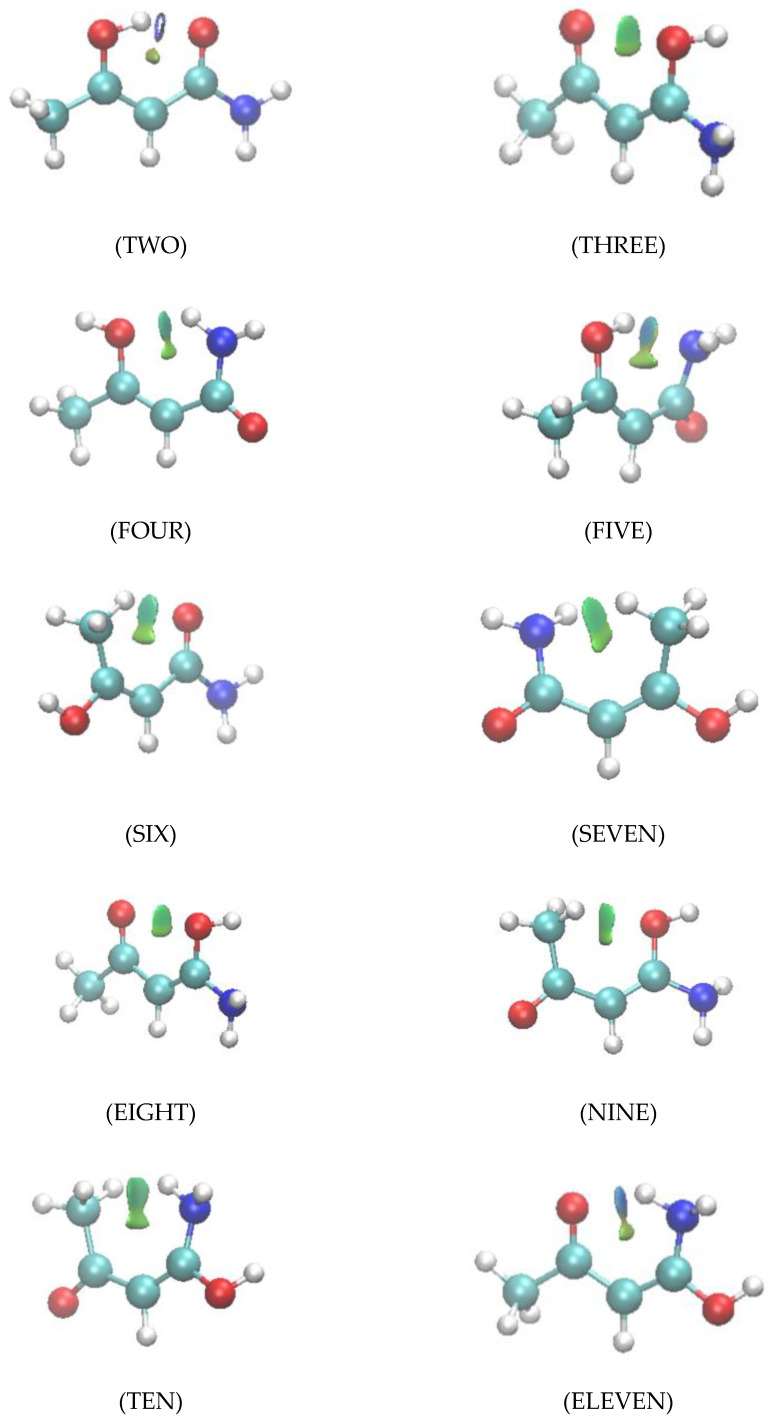
Panels display the isosurfaces for the noncovalent interactions in species TWO through ELEVEN. Species ONE is discussed in detail in the Methods and Software section below. Color coding identify regions of repulsive interaction as green and the attractive regions as blue.

Isosurfaces for the reduced density gradient characterizing the noncovalent interactions in all species are shown in Figure 7. The color coding identifies the O…O interaction in THREE and EIGHT (entirely green) as repulsive, despite the substantial density at the BCPs. In cases TWO and ELEVEN the portion of the NCI isosurface enclosing the BCP is blue (attractive), and the portion enclosing the RCP is green. The NCI enclosing surfaces for weakly interacting CH…O and CH…N systems are of more subtly varying hue. All these qualitative observations comport with our understanding of the relative strength of the hydrogen bonding.

### 2.5. Expression of Hydrogen Bonding in MP2/cc-pVTZ Computed Harmonic Vibrations

Hydrogen bonding is often expressed in the vibrational spectrum. Here we discuss the canonical frequencies which correspond to normal modes. These are in principle delocalized combinations of local modes, but in some cases the local modes are well isolated. These include OH, NH_2_, and CH_3_ group modes. See Table 3.

**OH Mode:** The reference OH stretches–uninfluenced by H bonding–fall in the set (THREE, SEVEN, and ELEVEN) and have values 3836, 3850, and 3842 respectively. EIGHT and TEN have OH modes at 3807, which may be associated with coupling of OH at a carbon also bearing NH_2_, both being uninvolved in intramolecular hydrogen bonding. The most drastically shifted OH stretches are for TWO to 3087 and FIVE to 3571, suggesting that the OH…O= interaction in TWO is stronger than the OH…NH_2_ interaction in FIVE, and that OH is not strongly engaged in hydrogen bonding in any other system.

**NH_2_ Modes:** NH stretching modes for most systems cluster in the range 3720–3780 for the asymmetric combination and 3600–3630 for the symmetric combination. High values for the differences Δν between asymmetric and symmetric stretching frequencies correspond to NH participation in H bonding for (especially) system ELEVEN (Δν = 308) and to a lesser extent for system ONE (Δν = 159). ONE and ELEVEN have the H-bonding structure (NH…O=) in common, but the larger shift in ELEVEN is easily attributed to the shorter N…O distance in ELEVEN (2.617 Å) than is found for ONE (2.789 Å). The next largest difference is for FOUR (Δν=151), which has an NH…OH interaction.

The NH stretching modes in EIGHT (Δν = 120) and NINE (Δν = 119) have minimal differences. In both cases the NH_2_ group is isolated from H bonding. The remaining systems have splitting ranging from 132 to 151. TWO (Δν = 147) and SIX (Δν = 144) have comparable splitting; for each, NH_2_ is attached to a carbonyl carbon. FIVE (132), SEVEN (135), and TEN (136) fall in a narrow range. In each of these, N is a hydrogen bond acceptor. THREE (with Δν = 138) is unique.

**CH stretches in the methyl group:** Methyl CH stretches in a C_3v_ environment include the A_1_ all-in-phase “breathing” mode and an E set of out-of-phase motions. In this low symmetry setting we can identify motions corresponding to those in high symmetry. The mode analogous to the A_1_ breathing is lowest in frequency, ranging from 3060 to 3090 for species ONE through ELEVEN. The former E stretching combinations, which split into in-phase and out-of-phase modes in the low symmetry environment, appear in ranges 3130–3180 and 3060–3090 respectively. There seems to be no pattern in these values indicating whether the methyl group can participate in CH…X interaction.

**CCC bend:** For THREE and EIGHT, for which no H bonding is recognizable and a repulsive NCI zone lies between the C=O and OH oxygens, the C-C=C bending modes have the lowest a frequency, 178. For other systems the C-C=C bend is our rough surrogate for hydrogen bond stretching. The highest bending mode frequencies are found for ELEVEN (242), SEVEN (247), TWO (246) and ONE (242). These have NH…O=, CH…NH_2_, OH…O=, and weakened NH…O= interactions respectively. Mixing with the methyl internal rotation sometimes makes the isolation of the CCC bend difficult; strong coupling is evident for SEVEN and other species with CH…X interaction.

Coupling of local modes within canonical normal modes complicates the interpretation of hydrogen bonding by inspection of vibrations. For example, the C=O and C=C stretching modes are strongly coupled in FOUR, but are weakly coupled in NINE and TEN. Furthermore, the COH bend is often strongly coupled to the CO stretch. as well. We expect that extraction of local modes from the canonical normal modes will simplify the discussion of H bonding and vibrations. This is accomplished by the Local Mode Analysis [41,42].

### 2.6. Local Mode Analysis

The Local Mode Analysis allowed identification of force constants for several diatomic stretching motions for all species ONE through ELEVEN. These included the two C=O stretches for ONE (Table 4) and the single C=O stretches for TWO through ELEVEN (Table 5). CO and OH stretches were also defined for TWO through ELEVEN. The three methyl CH stretches and the two NH stretches were chosen for all species. For systems which can plausibly be assigned hydrogen bonding structures, the X…HY stretches were included in the analysis.

Local CH stretches span a narrow range of frequencies, ca. 3100 to 3200 cm^−1^. There seems to be no great impact on these values even in systems that may have CH…O or CH…N interactions.

C=O stretches extend from 1650 to near 1700. The exception is species TWO at 1550, which has strong =O…HO hydrogen bonding. OH stretches which do not participate on hydrogen bonding have frequencies near 3800 to 3850 (THREE, SEVEN, EIGHT and NINE) while TWO and FIVE have seriously reduced OH stretching frequencies, in keeping with their participation in hydrogen bonding.

Species TWO, THREE, and SIX have both local NH force constants above 7.2 and both local frequencies above 3600. These systems do not involve the NH_2_ group in H bonding. ONE and ELEVEN engage one NH bond in hydrogen bonding which is reflected in one low NH stretching frequency (3564 and 3212). FOUR is a puzzling exception, with near-identical NH force contants. TEN shows both NH force constants of 6.8 and frequencies near 3500, and FIVE has its two modes with force constants near 7.1 and frequencies near 3579. EIGHT and NINE have one local mode with low frequency (below 3600) with the other mode higher than 3600. These values are consistent with an interaction between substituents OH and NH_2_ on an unsaturated carbon.

Yannacone et al. [18] established an empirical power law relation between density at a bond critical point and local force constants, of the form

*ln (k_HBOND_)* = A *ln(ρ_BCP_)* + B, (2)

We fit the computed local force constants for TWO and ELEVEN to this form, finding A = 0.740 and B = 0.934, and inferred the remaining values reported in Table 4. The sequence of H bond strength begins with the strongest interactions, found in TWO > ELEVEN > FIVE which have *k_LOCAL_* above 0.200. The central cluster includes ONE, FOUR, and SIX with NH…O=, >NH…OH, and CH…O= interactions. The weakest interactions (in descending order) are CH…N, CH…N, and CH…OH. It is interesting to see that the strongest CH…X interaction (with X a carbonyl oxygen) is comparable to NH…O interactions.

## 3. Discussion

**Figure 8:**
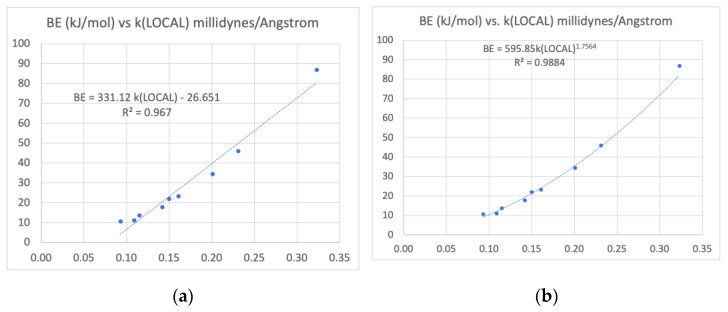
(**a**) Linear correlation between the local force constants *k_LOCAL_* and the Espinosa binding energy (BE) defined as BE = −V/2 where V is the potential energy density evaluated by QTAIM at the H bond’s Critical Point. (**b**) Correlation diagram with a power law fit. The rank order of H bond strengths agrees with the ordering predicted by *k_LOCAL_* and other measures, including the Kraka bond order *n* and the density itself at the BCPs. A linear fit is imperfect, but illustrates a strong relation between variables.

We have described the intramolecular interactions of conformations of acetoacetamide and isomers of its tautomer 2-oxopropanolamine by accurate *ab intio* calculations of relative energies and analysis of their density by AIM and NCI theories. Recasting the vibrational modes by the Cremer-Kraka local modes analysis allow reliable assignments of relative strengths of hydrogen bonds XH…Y with (X, Y) including (O, O), (O, N), (N, C), and (O, C) (Figure 8). Rank ordering of bond strength by measures bond order n, electron density *ρ* and potential energy density V, and also local force constants k produce an agreed sequence of bond strengths. In specific, ordering according to a bond energy BE estimate based on the potential energy density at the bond critical point agrees with ranking based on local force constants.

**Figure 9:**
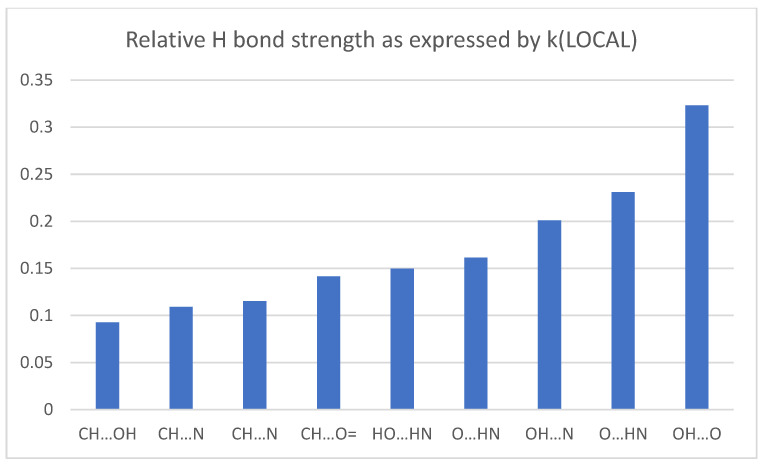
Local force constants (millidynes/Angstrom) for hydrogen bonds.

All measures agree that the H bond strength decreases in magnitude in the order shown in Figure 9, which shows the values of the local force constants for H bonds.

We expect that the analysis can be extended to many molecules of biological importance and to the description of other kinds of noncovalent interactions.

## 4. Materials and Methods

We employed extrapolation techniques to arrive at accurate relative energies. The Atoms in Molecules analysis of the computed density, and its extension to the reduced gradient description of non-covalent interactions allowed one perspective on the relative strength of our system’s hydrogen bonds. An alternative view is provided by analysis of the vibrational spectrum and its canonical (normal) modes. Recovery of local vibrational modes and their associated frequencies allows more direct discussion properties of the hydrogen bonds.

### 4.1. Extrapolation Techniques

A well-established method for obtaining highly accurate energies (including energy differences among conformational isomers) is the extrapolation of energies obtained in a sequence of more and more flexible basis sets [35]. Such techniques depend on the behavior of energy values obtained in a sequence of basis sets embracing a series of values of the angular momentum. The Dunning basis sets of general form cc-pV*L*Z, with maximum angular momentum *L* = 2, 3, 4,… are most often used [36,37]. The Hartree-Fock energy is known to scale exponentially, while the correlation energy follows a *L*^−3^ inverse-cubic scaling for sufficiently large *L* [38]. Simple schemes based on this behavior are often usefully accurate. We employ the formulas shown below.

(3) $$E_{HF}\left(\infty \right)=\frac{{\left(E_{HF}\left(L-2\right)-E_{HF}\left(L-1\right)\right)}^{2}}{\left(E_{HF}\left(L-2\right)-2E_{HF}\left(L-1\right)-E_{HF}\left(L\right)\right)}$$

(4) $$E_{CORR}\left(\infty \right)=\frac{\left(L^{3}E_{CORR}\left(L\right)-{\left(L-1\right)}^{3}E_{CORR}\left(L-1\right)\right)}{\left(4L^{3}-{\left(L-1\right)}^{3}\right)}$$

Here *L* denotes the maximum angular momentum in the extrapolation, so if we use cc-pVQZ as the largest basis, *E_HF_*(*L*) and *E_CORR_(L)* refer to the energies obtained with that basis, *L −* 1 to cc-pVTZ, and *L −* 2 to cc-pVDZ.

A very recent refinement in the extrapolation of the correlation energy has been reported by Lesiuk and Jeziorski [39] which employs *E_CORR_* for *L* and *L −* 1. Defining the constant *a* by

(5) $$a=L^{4}\left(E_{CORR}\left(L\right)-E_{CORR}\left(L-1\right)\right)$$

The limit of the correlation energy is

(6) $$E\left(\infty \right)=E_{CORR}\left(L\right)+a\left[\frac{\pi^{4}}{90}-\overset{L}{\underset{l=1}{\sum }}l^{-4}\right]$$

We found that the two-point Zeta-extrapolation lowered the final estimate of total electronic energy by 15 millihartrees. However, the Zeta-extrapolation had only a very small impact (<0.2 kJ/mol.) on relative energies obtained with the simpler scheme.

We do not employ any correction intended to overcome basis set superposition error, since it appears that the complete basis set limit of energies is only very slightly altered by its inclusion [35]. The fact that we are studying intramolecular effects may further discount its significance.

Extrapolation is also an important part of well-established thermochemical schemes, which include the CBS series [33,40], the Gn series [41], and more demanding schemes such as Wn [42,43] and HEAT [44]. These methods include empirical corrections lacking in our calculations. To complement our extrapolations we performed G4 calculations, finding that the G4 estimates of relative electronic energies of species in question were in disagreement with our values by no more than 1–2 kJ/mol.

### 4.2. Atoms in Molecules

The atoms in Molecules (AIM) theory of Bader [13,27,45] is constituted of a description of the molecular charge density *ρ*, its gradient and Hessian, and related quantities including kinetic and potential energy densities. Extreme values of the density demark significant regions in the molecular charge distribution. Points with ∇*ρ* = 0 may be described as quantum atoms, bond critical points (BCPs), ring critical points (RCPs) and cage critical points (CCPs) depending on the sign structure of the set of eigenvalues of the Hessian matrix of the density at those points. A locus of points with zero density gradient connecting atom centers through a BCP is termed a bond path. These paths very often correspond to intuitive ideas of chemical bonds. However serious complications are often encountered in the chemical interpretation of bond paths and critical points for weak interactions, as mentioned already [22,23,24,25,26,27,28].

The AIM analysis was applied to H bonding from the beginning. Koch and Popelier [46] developed criteria for judging H-bonding in AIM context. They include:

1. Topology: There should appear a bond path between atoms considered to be linked by a H bond. This path should contain a BCP.
2. Density: At this point the density should have a “reasonable” value, about an order of magnitude smaller than the values for covalent bonds or ca 0.01 atomic units, with a range up to 0.035 atomic units. There is a correlation between the BCP density and *inter*molecular attraction.
3. Laplacian: ∇^2^*ρ* must be positive, with values in an approximate range 0.02 to 0.10 atomic units. This sign suggests a depletion of charge at the BCP, and is characteristic of an interaction between closed shells [13,21].
4. H atom charge: q(H) involved in a hydrogen bond XH…Y should be substantially smaller than q(H) for H atoms not participating in a H bond but with the same X atom in XH. Here q(H) is the charge in the basin containing the nuclear attractor for H atom.

Further criteria include Atomic interpenetration; Destabilization of the H atom; Diminished atomic polarizability for the H atom; and Decreased volume of the H atom. In this work we confine our attention to the first four criteria, as has been the practice in most studies of AIM-based description of intramolecular H bonding in conformational isomers.

The delocalization index δ(A, B), in contrast to many of the parameters of AIM theory, refers to the two-electron density matrix, and is a measure of electrons shared between two basins A and B. A perfectly covalent bond would have a value for δ(A, B) near the integer associated with a Lewis structure, but an ionic component to bonding reduces the value of δ(A, B) [47,48]. The parameter has been used to discuss the strengths of intermolecular hydrogen bonds [48].

### 4.3. Non-Covalent Interactions and the Reduced Density Gradient

The measure *s(r)* for non-covalent interaction (NCI) was introduced by Johnson et al. [49,50] The reduced (dimensionless) density gradient *s* (also called RDG) is defined as

(7) $$s\left(r\right)=\frac{\left|\nabla \rho \left(r\right)\right|}{2{\left(3\pi^{2}\right)}^{1/3}\rho \left(r\right)r^{4/3}}$$

The RDG has been applied to the description of H-bond strength [51]. A useful description of the RDG, its graphical characterization, and its interpretation is given by Contreras-Garcia, et al. [52].

**Figure 10:**
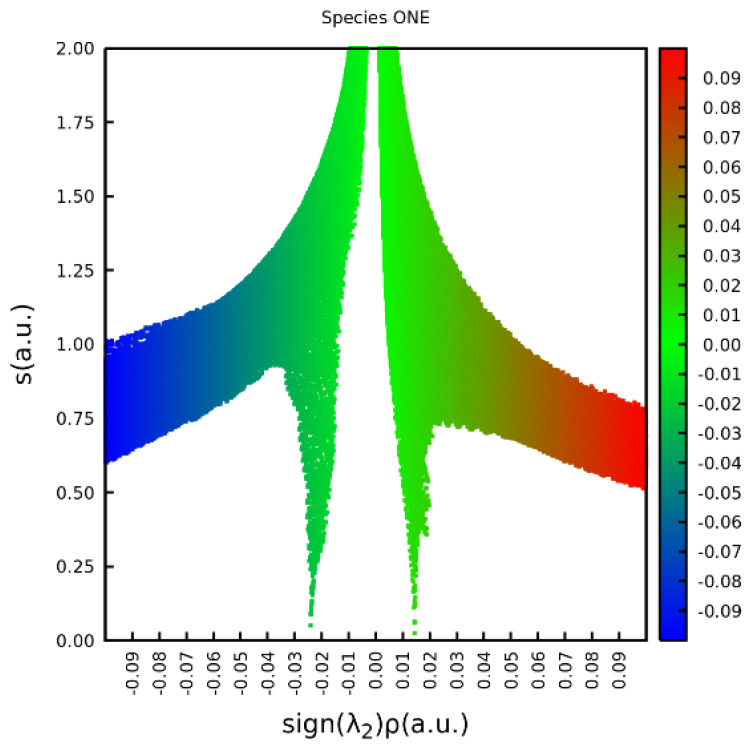
Reduced density gradient *s* vs. the density signed by the second eigenvalue of the Hessian for species ONE. A positive sign indicates a repulsive non-covalent interaction (often found in the center of a closed ring), and a negative value denotes attraction (H bonding in this case).

Two NCI graphic realizations are useful. A two-dimensional plot of *s* vs. the density (given the sign of the second eigenvalue of the Hessian **H**) will produce a broad sweep from one extreme at small density but large *s* through an extreme and large *s* and small density and then to a second extreme of small density and small *s*. (Figure 6) This is characteristic of an exponentially-decaying density. Superimposed on this sweep may be spikes in *s* extending to values near zero at modest values of density. The high-density (but low *s*) regions near nuclei are far to the right or left of the range of densities shown. The low-density region is located primarily in Cartesian space close to the density tails for which *s* is large. However, *s* can be small precisely where the density is disturbed by non-covalent interactions, such as van der Waals/dispersion, electrostatic forces, and hydrogen bonds. Figure 10 is the plot for TWO, the most stable species, which contains a strong -OH…O= hydrogen bond with its corresponding critical point, and a closed ring marked by a ring critical point. Two such spikes are shown, at about +0.015 and −0.025 signed density units. Generally spikes fall into three types: (i) negative values of the signed density indicative of attractive interactions, such as dipole-dipole or H-bonding, (ii) positive signed density indicating non-bonding interactions, such as electrostatic or steric repulsion in the ring/cage, and (iii) values near zero indicating very weak interactions, such as van der Waals interaction.

**Figure 11:**
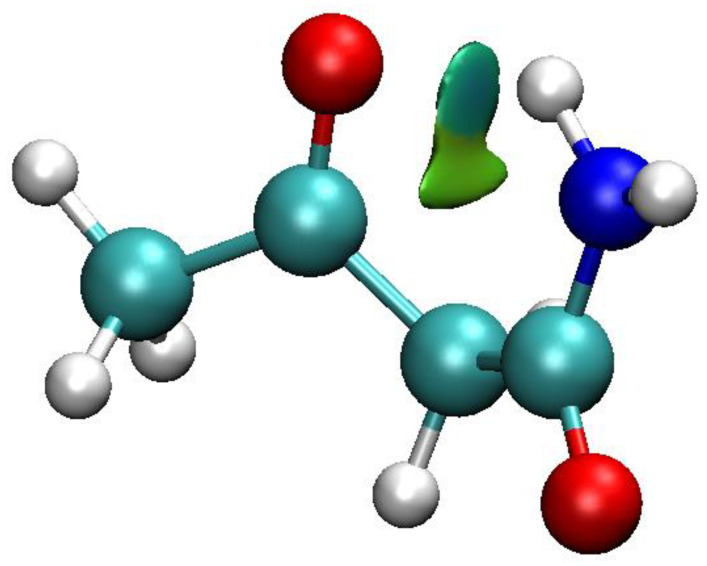
The NCI isosurfaces for ONE make evident the repulsive noncovalent interaction at the RCP interior to the ring (green coded) and the attractive noncovalent interaction at the BCP for the hydrogen bond (blue).

Figure 11 shows the isosurface obtained by mapping data from Figure 10 into three-dimensional space. The blue region shows an attractive interaction (according to the reduced density gradient criterion) at the Bond Critical Point along the Path O…H. At a Ring Critical Point a green isosurface encloses a region of repulsive non-covalent interaction.

### 4.4. Canonical Vibrational Spectra and Local Mode Analysis

Computed vibrational frequencies are generally recovered as eigenvalues of the matrix of second derivatives of the energy with respect to mass-weighted displacement coordinates, in the formulation developed by Wilson and coworkers [53]. The associated eigenvectors, normal modes, are generally delocalized. In the study of the strength of hydrogen bonding discussion is eased by the introduction of local force constants without reference to atomic masses. A means of recovery of local force constants from vibrational data was developed by Cremer and coworkers [54]. An overall review is provided by Kraka, Zou, and Tao [55]. The hydrogen bond, among many other kinds of noncovalent interaction has been analyzed [56,57,58].

### 4.5. Software

Gaussian 09 [59] and 16 [60] produced optimized structures and the extrapolation sequence of RHF, MP2, and coupled-cluster energies. Atoms-in-Molecules results were obtained with AIMALL [61]. NCIPLOT was obtained by download from the Contreras-Garcia group [62] and LMODEA software for the local modes analysis [63] was provided by the Kraka group.

## Figures and Tables

**Table 1:** Species exhibiting categories of interaction and their relative energies (kJ/mol.).

No H Bonding	CH…O or CH…N	OH…O, OH…N, NH…O
THREE (+62) EIGHT (+101)	SIX (+43) SEVEN (+38) NINE (+91) TEN (61)	ONE (4.0) TWO (0.0) FOUR (+42) FIVE (+38) ELEVEN (+48)

**Table 2:** Topological data for critical points associated with non-covalent interaction in species ONE–ELEVEN.

Species	Interaction	*ρ*	∇^2^*ρ*	H	V	Q(H)	Order	DI
ONE	C=O…HN	0.0241	0.0931	0.00640	−0.0177	0.4636	0.164	0.0642
TWO	-OH…O=	0.0615	0.1158	−0.01860	−0.0661	0.3273	0.221	0.1254
THREE	NCI	0.0110	0.0564		−0.0080	0.5876		
FOUR	HO…HN	0.0218	0.0974	0.00390	−0.0167	0.4485	0.158	0.0541
FIVE	−OH…NH_2_	0.0324	0.0870	−0.00220	−0.0262	0.3661	0.180	0.0831
SIX	CH…O=	0.0202	0.0782	0.00300	−0.0135	0.1164	0.155	0.0715
SEVEN	CH…NH2	0.0140	0.0517	0.00230	−0.0084	0.0604	0.138	0.0531
EIGHT	NCI	0.0133	0.0560		−0.0081	0.5978		
NINE	CH…OH	0.0114	0.0532	0.00260	−0.0081	0.0140	0.129	0.0384
TEN	CH…N	0.0137	0.0511	0.00220	−0.0083	0.0434	0.142	0.0276
ELEVEN	=O…HN	0.0391	0.1192	−0.00260	−0.0350	0.5022	0.191	0.1003

***ρ*** = density; ∇^2^***ρ*** = Laplacian of density; **G** = Kinetic energy density; **V** = potential energy density; **Q(H)** = integral of charge in H atom basin. Order = Kraka definition of bond order and DI = delocalization index for H atom and its partner in the species’ putative hydrogen bond. Analysis by AIMALL of density computed with ωB97XD/cc-pVTZ.

**Table 3:** anonical harmonic frequencies (in cm^−1^) for normal modes of all systems, computed by MP2/cc-pVTZ.

Species	NH_2_ Stretches		Double Bond Stretches		OH Stretches
ONE	3725a	3566s	1790 ^1^	1757 ^2^	None
TWO	3777a	3630s	1725	1710	3087
THREE	3747a	3609s	1782	1748	3836
FOUR	3777a	3626s	1773	1736	3853
FIVE	3659s	3537s	1780	1704	3571
SIX	3766a	3622s	1771	1742	3842
SEVEN	3726a	3591s	1731	1772	3850
EIGHT	3679a	3559s	1763	1714	3807
NINE	3673a	3554s	1712	1750	3803
TEN	3726a	3591s	1772	1731	3850
ELEVEN	3655a	3347s	1685	1742	3818
**Species**	**CH_3_ Stretches** ^4^			**CCC Bend** ^5^	**CO Stretch**
ONE	3208s	3157a	3076b	236	None
TWO	3198s	3164a	3084b	246	1356
THREE	3202s	3155a	3074b	178	1385
FOUR	3198a	3143s	3063b	226	1347
FIVE	3203s	3162a	3084b	226	1317
SIX	3213s	3135a	3062b	226	1348, 1267
SEVEN	3213s	3132a	3063b	193	1351, 1273
EIGHT	3202s	3155a	3074b	178	1467
NINE	3205s	3175a	3089b	207	1435
TEN	3214a	3132s	3063b	194	1273
ELEVEN	3204s	3161a	3079b	242	1367

^1^ Predominantly C=O. ^2^ Predominantly C=C. NH_2_ stretches are either antisymmetric (a) or symmetric (s) combinations of local NH motions ^4^ CH stretches descend in symmetry from the C_3v_ limit; the A_1_ mode can be recognized as “breathing” breathing (b) and components of the E pair are the symmetric (s) and antisymmetric (a) modes. ^5^ The C-C-C bend serves as a surrogate for the hydrogen bond stretch.

**Table 4:** For species ONE, local force constants (millidynes/Å) and associated frequencies (cm^−1^).

Species	C=O	C=O′	NH	NH′	C-H	C-H′	C-H″	H…O
ONE	11.7 1700	11.7 1699	7.4 3654	7.0 3564	5.5 3181	5.3 3116	5.3 3112	0.161 537

**Table 5:** For species TWO–ELEVEN, local force constants (millidynes/Å) and associated frequencies (cm^−1^).

Species	C=O	C-O	NH	NH′	C-H	C-H′	C-H″	O-H	X…Y ^1^
TWO	9.7 1552	6.5 1273	7.4 3651	7.0 3670	5.5 3169	5.4 3130	5.4 3130	4.3 2801	0.323 760
THREE	11.7 1701	6.2 1237	7.3 3641	7.3 3630	5.5 3174	5.3 3103	5.3 3103	8.2 3829	NCI
FOUR	11.4 1678	5.6 1177	7.6 3666	7.6 3661	5.5 3178	5.3 3099	5.3 3099	8.3 3848	0.150 764
FIVE	11.8 1708	6.0 1214	7.1 3578	7.0 3560	5.5 3176	5.4 3129	5.4 3127	6.7 3475	0.201 712
SIX	11.3 1671	5.8 1202	7.4 3657	7.4 3644	5.6 3186	5.3 3100	5.3 3100	8.2 3836	0.142 732
SEVEN	11.6 1694	5.8 1195	7.2 3616	7.3 3631	5.6 3191	5.3 3098	5.3 3098	8.2 3844	0.109 612
EIGHT	11.6 1694	5.8 1253	7.0 3557	7.2 3610	5.5 3176	5.3 3113	5.3 3113	8.0 3757	NCI
NINE	11.2 1666	6.0 1219	7.0 3553	7.2 3606	5.5 3172	5.4 3127	5.4 3127	8.0 3789	0.093 711
TEN	11.2 1664	5.8 1197	6.8 3511	6.8 3598	5.2 3176	5.0 3022	5.2 2995	7.8 3728	0.115 688
ELEVEN	9.9 1566	6.0 1219	7.2 3594	5.7 3212	5.2 3176	5.2 3076	5.2 3076	8.1 3806	0.231 804

^1^ Atoms linked by the hydrogen bond. Local force constants are based on computed values for TWO and ELEVEN, which provided parameters for a power law fit to BCP densities. Local frequencies are inferred from scaling to the square root of local force constants.

## Data Availability

Not applicable.

## Supplementary Materials

The following are available online, which includes: Electronic energies used in extrapolation; Cartesian coordinates of all species; AIM figures with bond paths, line critical points and associated values of the Laplacian of the electron density.

## Sample Availability

Samples of the compounds Not applicable; no chemical material is used in this research.
