# Supplementary material for: Focal Point Evaluation of Energies for Tautomers and Isomers for 3-hydroxy-2-butenamide: Evaluation of Competing Internal Hydrogen Bonds of Types -OH…O=, -OH…N, -NH…O=, and CH…X (X=O and N)
Source: Molecules. 2021 Apr 30;26(9):2623. doi: 10.3390/molecules26092623 (PMC8124485; doi:10.3390/molecules26092623)
Supplement: Supplementary file 1 [file molecules-26-02623-s001.zip › SI-geometryandAIM.pdf]

Supplementary Information (Geometry)

Table of Contents

SYSTEM ONE 2

SYSTEM TWO 3

SYSTEM THREE 3

SYSTEM FOUR 4

SYSTEM FIVE 6

SYSTEM SIX 7

SYSTEM SEVEN 8

SYSTEM EIGHT 9

NINTH EXAMPLE 10

SYSTEM TEN 11

SYSTEM ELEVEN 12

System ONE

NUMBERING for ONE

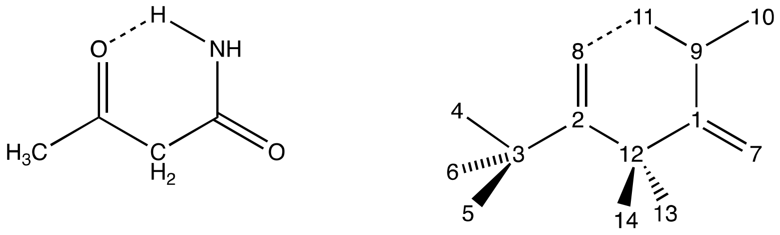

1 C1	-1.3491	-0.2156	0.0178	C
2 C2	1.2093	0.1308	0.0721	C
3 C3	2.4072	-0.7002	-0.2894	C
4 H4	3.2361	-0.0614	-0.5757	H
5 H5	2.1450	-1.3669	-1.1116	H
6 H6	2.6884	-1.3315	0.5535	H
7 O7	-2.1703	-1.0534	-0.3189	O
8 O8	1.2146	1.3482	-0.0281	O
9 N9	-1.5658	1.1235	-0.0146	N
10 H10	-2.3966	1.4356	-0.4869	H
11 H11	-0.7652	1.7336	0.0471	H
12 C12	0.0030	-0.6286	0.5915	C
13 H13	0.1067	-1.6971	0.4301	H
14 H14	-0.0301	-0.4538	1.6704	H

OH...N angle = 131°; N...O distance = 2.789 Å

SYSTEM TWO

NUMBERING for TWO

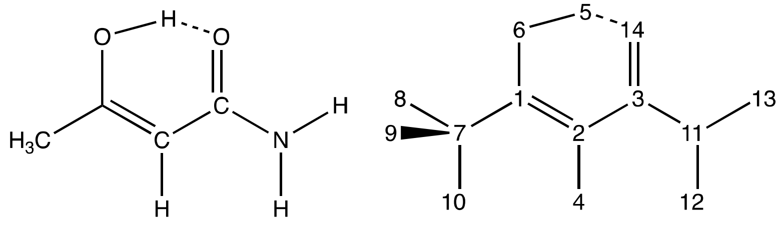

1 C1	1.1679	-0.0705	0.0003	C
2 C2	-0.0054	-0.7575	-0.0073	C
3 C3	-1.2628	-0.0311	-0.0040	C
4 H4	0.0109	-1.8351	-0.0197	H
5 H5	0.2587	1.5453	0.0028	H
6 O6	1.2197	1.2584	0.0073	O
7 C7	2.5078	-0.7195	0.0012	C
8 H8	3.0645	-0.4049	0.8827	H
9 H9	3.0700	-0.3954	-0.8734	H
10 H10	2.4225	-1.8019	-0.0050	H
11 N11	-2.4053	-0.7683	-0.0315	N
12 H12	-2.3779	-1.7539	0.1480	H
13 H13	-3.2714	-0.2776	0.1026	H
14 O14	-1.3180	1.2134	-0.0022	O

OH...O angle = 151°; O...O distance = 3.538 Å

SYSTEM THREE

NUMBERING for THREE

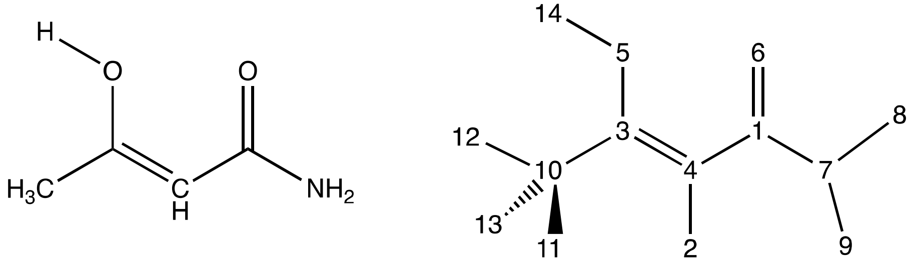

1 C1	-1.3359	0.0434	-0.0285	C
2 H2	-0.0391	-1.7351	-0.1183	H
3 C3	1.1689	-0.0649	0.0023	C
4 C4	-0.0391	-0.6578	-0.0510	C
5 O5	1.2971	1.2792	0.0882	O
6 O6	-1.4834	1.2539	-0.0865	O
7 N7	-2.4169	-0.8174	0.0136	N
8 H8	-3.2967	-0.3752	0.2206	H
9 H9	-2.2852	-1.7402	0.3865	H
10 C10	2.4479	-0.8375	-0.0261	C
11 H11	3.0513	-0.5323	-0.8826	H
12 H12	2.2643	-1.9049	-0.0955	H
13 H13	3.0267	-0.6418	0.8782	H
14 H14	2.2367	1.4872	0.1216	H

O...O distance = 2.787 Å

SYSTEM FOUR

NUMBERING for FOUR

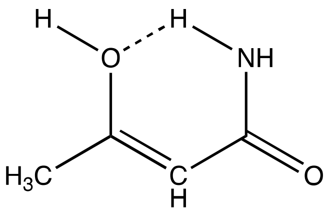

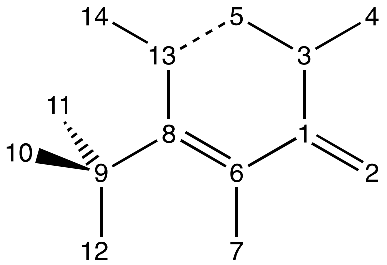

1 C1	1.4076	-0.2178	-0.0026	C
2 O2	2.3605	-0.9897	0.0260	O
3 N3	1.5654	1.1340	-0.0441	N
4 H4	2.5019	1.4832	0.0480	H
5 H5	0.7797	1.7510	0.0378	H
6 C6	0.0240	-0.7420	-0.0146	C
7 H7	-0.0283	-1.8188	-0.0256	H
8 C8	-1.1416	-0.0757	-0.0004	C
9 C9	-2.4764	-0.7417	-0.0052	C
10 H10	-3.0446	-0.4620	0.8838	H
11 H11	-3.0503	-0.4363	-0.8819	H
12 H12	-2.3703	-1.8215	-0.0212	H
13 O13	-1.1769	1.2961	0.0203	O
14 H14	-2.0963	1.5786	0.0346	H

O…HN angle = 129°; N…C distance = 2.746 Å

SYSTEM FIVE

NUMBERING for FIVE

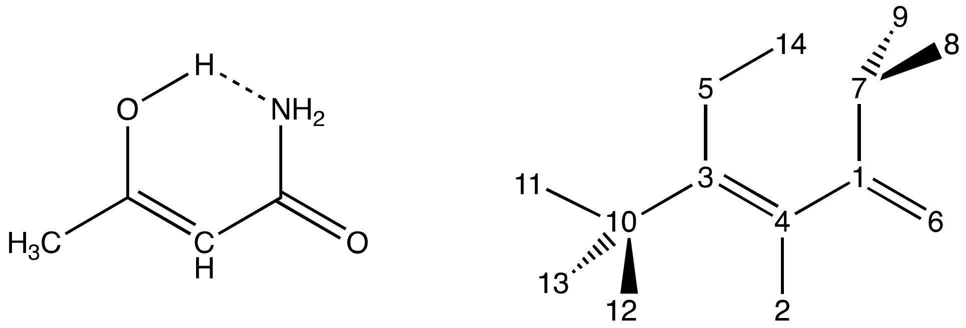

1 C1	1.1439	-0.0462	-0.0029 C
2 C2	0.0228	-0.7783	0.1943 C
3 H3	0.1266	-1.8386	0.3559 H
4 H4	0.2435	1.5542	-0.4421 H
5 O5	1.1690	1.2652	-0.3243 O
6 C6	2.5174	-0.6156	0.0806 C
7 H7	3.1008	-0.0582	0.8123 H
8 H8	3.0162	-0.5119	-0.8820 H
9 H9	2.4905	-1.6631	0.3629 H
10 O10	-2.2874	-0.9775	-0.3051 O
11 N11	-1.5009	1.1067	0.2421 N
12 H12	-2.4756	1.3703	0.1846 H
13 H13	-1.0735	1.4338	1.0988 H
14 C14	-1.3467	-0.2825	0.0363 C

OH...N angle = 138°; O...C distance = 2.735 Å

SYSTEM SIX

NUMBERING for SIX

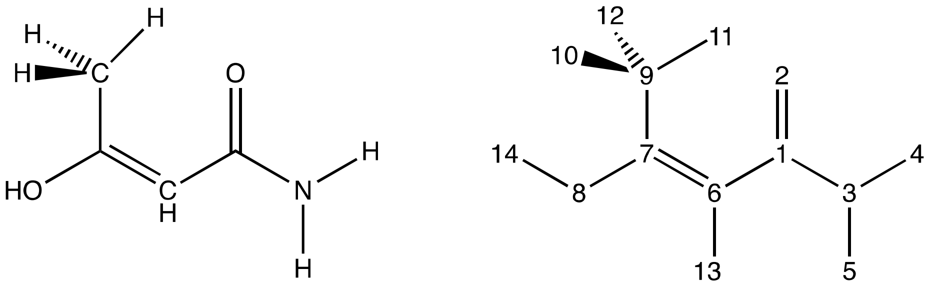

1 C1	-1.3070	0.0688	-0.0142	C
2 O2	-1.4014	1.2918	-0.0393	O
3 N3	-2.4288	-0.7231	-0.0103	N
4 H4	-3.2996	-0.2507	0.1592	H
5 H5	-2.3617	-1.6875	0.2567	H
6 C6	-0.0563	-0.7080	-0.0278	C
7 C7	1.1817	-0.1833	-0.0011	C
8 O8	2.2282	-1.0579	-0.0129	O
9 C9	1.5457	1.2594	0.0404	C
10 H10	2.1357	1.5121	-0.8425	H
11 H11	0.6529	1.8730	0.0656	H
12 H12	2.1573	1.4568	0.9227	H
13 H13	-0.1248	-1.7867	-0.0614	H
14 H14	3.0438	-0.5469	0.0057	H

CH...O angle = 130°; N...C distance = 2.948 Å

SYSTEM SEVEN

NUMBERING for SEVEN

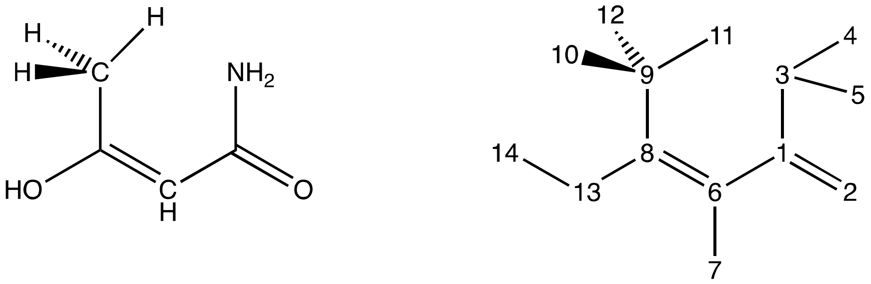

1 C1	-1.3763	-0.2084	-0.0124	C
2 O2	-2.2975	-0.8801	0.4283	O
3 N3	-1.5541	1.1182	-0.3642	N
4 H4	-2.5229	1.3942	-0.3998	H
5 H5	-0.9789	1.4734	-1.1084	H
6 C6	-0.0233	-0.7704	-0.1812	C
7 H7	0.0012	-1.8336	-0.3713	H
8 C8	1.1568	-0.1475	-0.0159	C
9 C9	1.4049	1.2668	0.3948	C
10 H10	1.8805	1.8228	-0.4152	H
11 H11	0.4888	1.7697	0.6810	H
12 H12	2.0877	1.2756	1.2465	H
13 O13	2.2852	-0.8963	-0.2065	O
14 H14	3.0490	-0.3613	0.0298	H

CH…N angle = 119°; N…C distance = 3.058 Å

SYSTEM EIGHT

NUMBERING for EIGHT

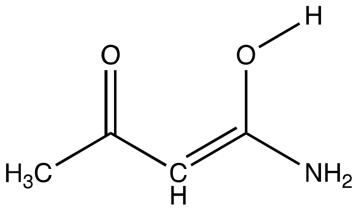

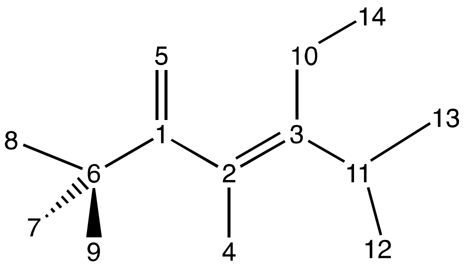

1 C1	-1.2902	0.1028	0.0017	C
2 C2	-0.0378	-0.6513	0.0628	C
3 C3	1.2045	-0.1137	0.0198	C
4 H4	-0.0957	-1.7281	0.1222	H
5 O5	-1.3641	1.3239	-0.0444	O
6 C6	-2.5385	-0.7569	-0.0031	C
7 H7	-2.5316	-1.4269	-0.8630	H
8 H8	-3.4161	-0.1196	-0.0413	H
9 H9	-2.5715	-1.3766	0.8932	H
10 O10	1.4195	1.2127	0.0693	O
11 N11	2.3666	-0.8615	-0.1273	N
12 H12	2.2019	-1.8561	-0.1318	H
13 H13	3.0848	-0.6160	0.5404	H
14 H14	2.2902	1.3758	-0.3146	H

O…O distance = 2.788 Å

NINTH EXAMPLE

NUMBERING for NINE

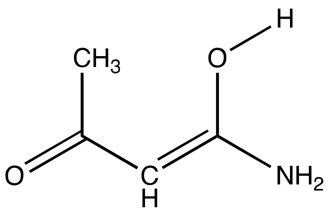

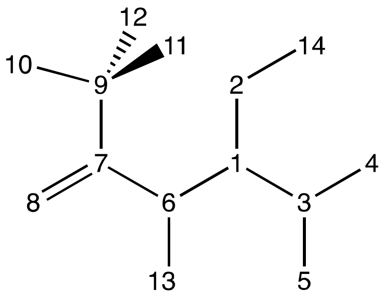

1 C1	-1.6131	1.2279	0.0843	C
2 H2	-2.6833	1.3852	0.1747	H
3 H3	-1.0927	1.6597	0.9366	H
4 H4	-1.2359	1.7306	-0.8042	H
5 C5	-1.3635	-0.2585	-0.0026	C
6 C6	0.0035	-0.7789	-0.0323	C
7 H7	0.0736	-1.8563	-0.0678	H
8 O8	-2.3045	-1.0463	-0.0451	O
9 C9	1.1654	-0.0903	-0.0190	C
10 O10	1.2244	1.2601	-0.1296	O
11 N11	2.4173	-0.6721	0.1463	N
12 H12	2.3771	-1.6771	0.2209	H
13 H13	3.0810	-0.3897	-0.5624	H

14	H14	2.0459	1.5405	0.2935	H

CH…O angle = 129°; N…C distance = 2.948 Å

SYSTEM TEN

NUMBERING for TENprime

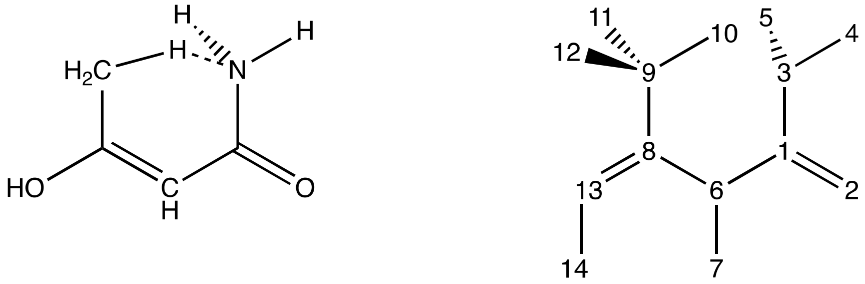

1	C1	1.3890	-0.2040	-0.0072	C
2	O2	2.3296	-0.9093	0.3447	O
3	N3	1.5515	1.1557	-0.2766	N
4	H4	2.5351	1.4054	-0.3651	H
5	H5	0.9732	1.5230	-1.0275	H
6	C6	0.0176	-0.7592	-0.1449	C
7	H7	0.0165	-1.8476	-0.2835	H
8	C8	-1.1827	-0.1324	-0.0044	C
9	C9	-1.4470	1.3081	0.3231	C
10	H10	-0.5406	1.8198	0.6665	H
11	H11	-1.8544	1.8295	-0.5606	H
12	H12	-2.2197	1.3486	1.1073	H

13 O13	-2.3646	-0.8053	-0.1267	O
14 H14	-2.1521	-1.7270	-0.3439	H

CH...N angle = 128°; N...C distance = 3.061 Å

SYSTEM ELEVEN

NUMBERING for ELEVEN (ZEOPTFREQMP2TZ)

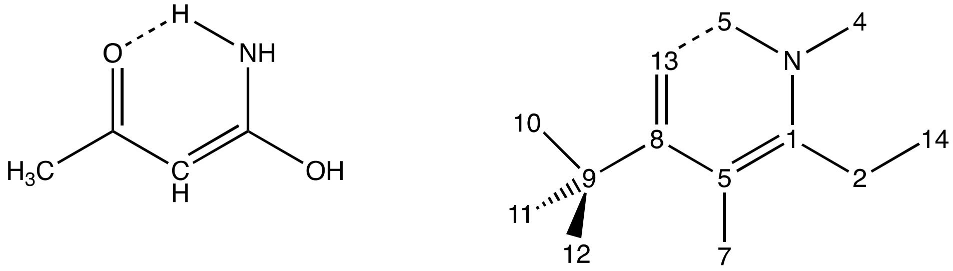

1 C1	1.2044	-0.1691	-0.0138	C
2 O2	2.2955	-0.9635	0.0679	O
3 N3	1.4245	1.1694	-0.0791	N
4 H4	2.1892	1.5226	0.4739	H
5 H5	0.5403	1.6817	-0.0192	H
6 C6	-0.0345	-0.7385	-0.0591	C
7 H7	-0.0987	-1.8126	-0.1107	H
8 C8	-1.2305	0.0636	-0.0067	C
9 C9	-2.5378	-0.6913	0.0282	C
10 H10	-3.3638	0.0118	0.0616	H
11 H11	-2.6270	-1.3227	-0.8554	H
12 H12	-2.5720	-1.3437	0.9002	H

13	O13	-1.2343	1.3056	0.0102	O
14	H14	3.0614	-0.4489	-0.2133	H

NH…O= angle = 134°; N…O distance = 2.617 Å

Supplementary Information – AIM

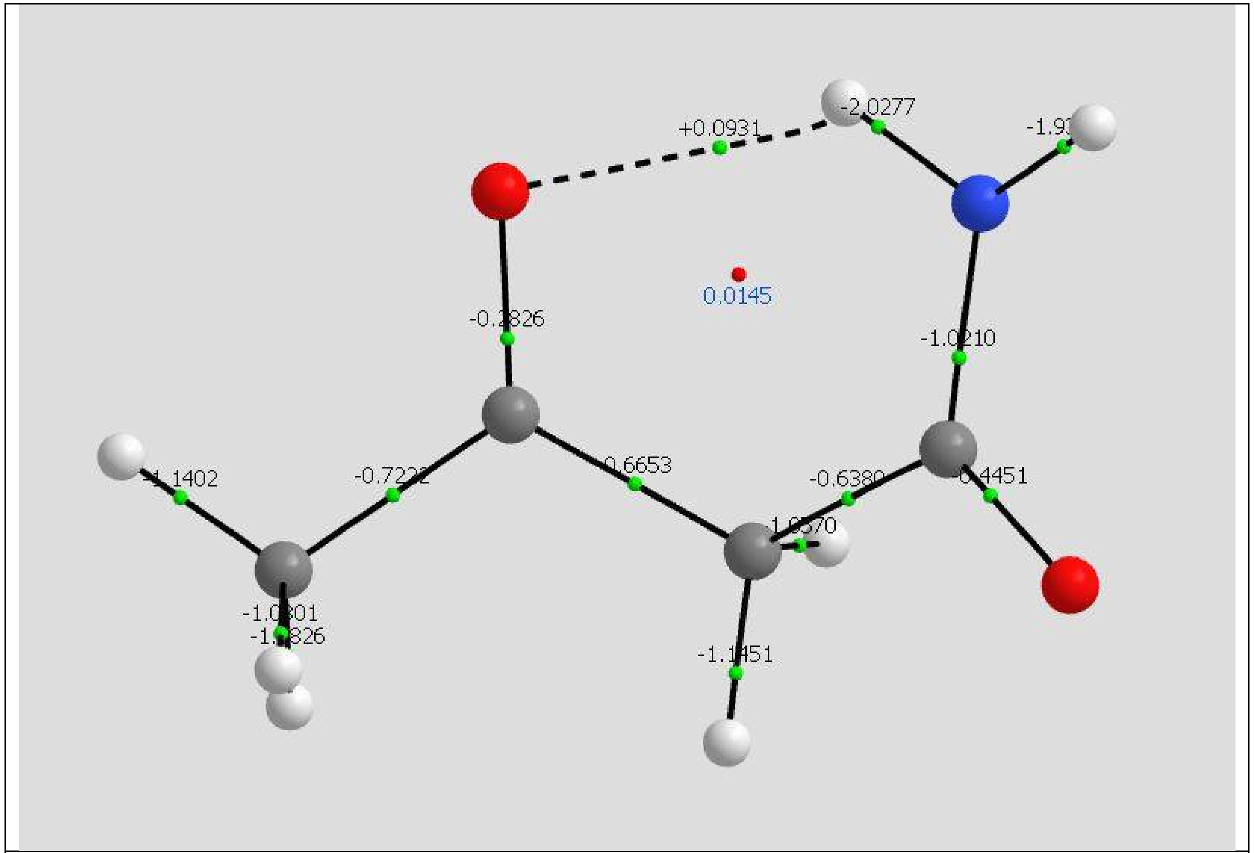

Species ONE: Laplacian of the density at Bond Critical Points, including the >NH…O= hydrogen bond

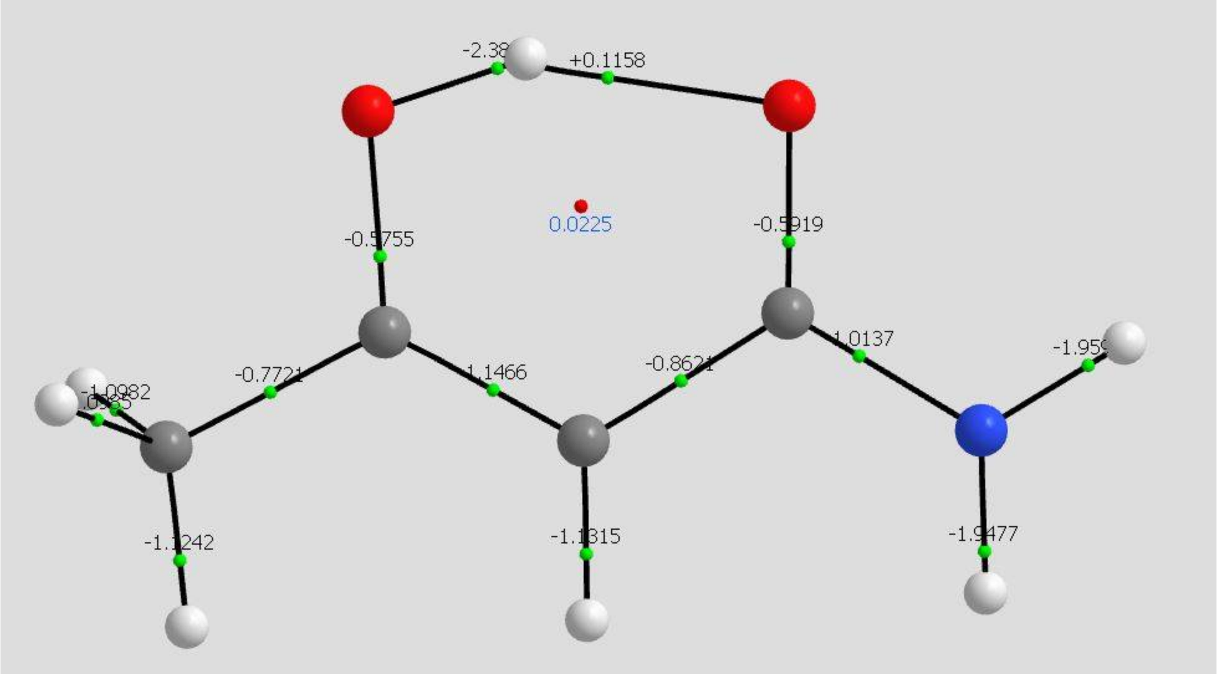

Species TWO: Laplacian of the density at Bond Critical Points, including the =O…HO- hydrogen bond

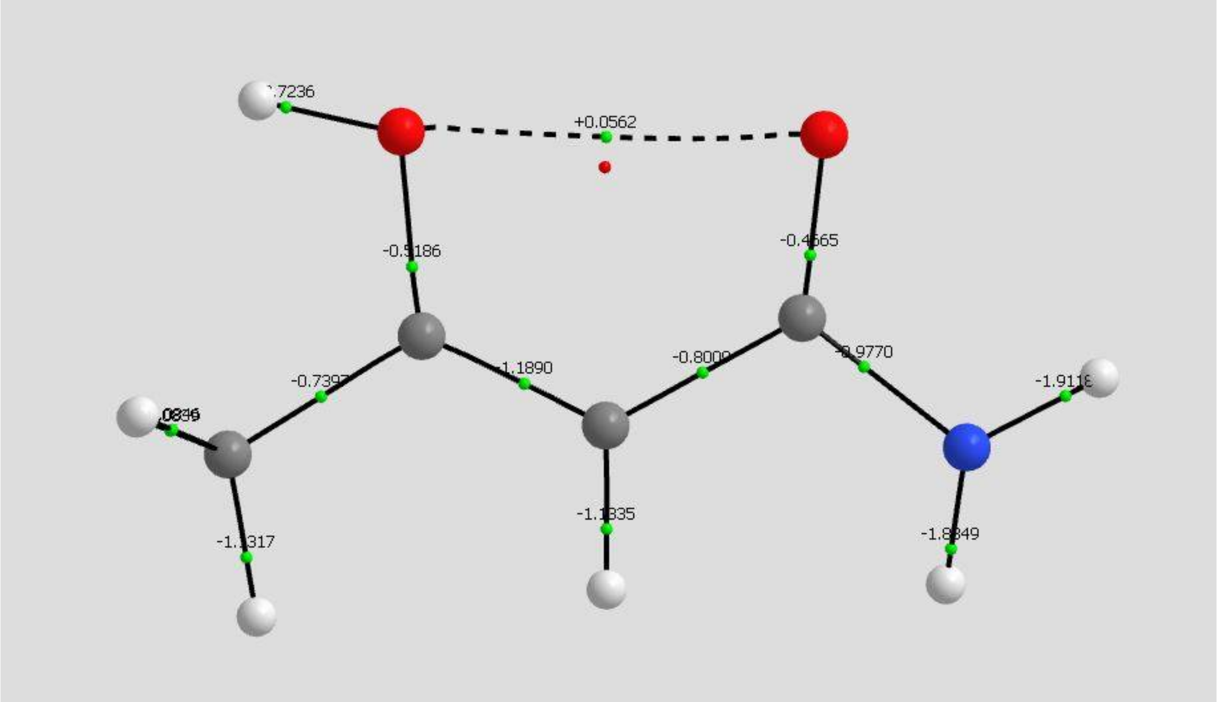

Species THREE has no recognizable H bond, but there is a non-covalent interaction between O atoms

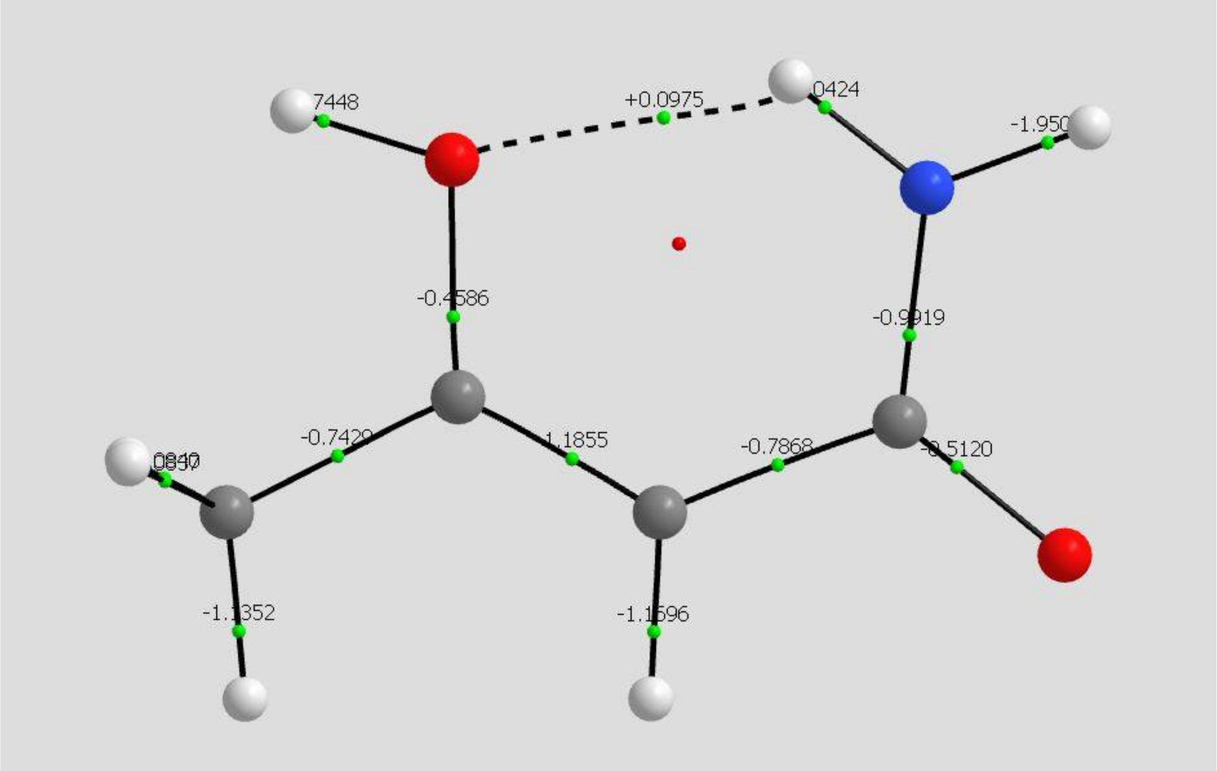

Species FOUR: Laplacian of the density at Bond Critical Points, including the HO…HN- hydrogen bond

Figure 1: Topology of bonding in species ONE, TWO, THREE, and FOUR. Numerical values of the Laplacian are shown at Bond Critical Points (green dots)

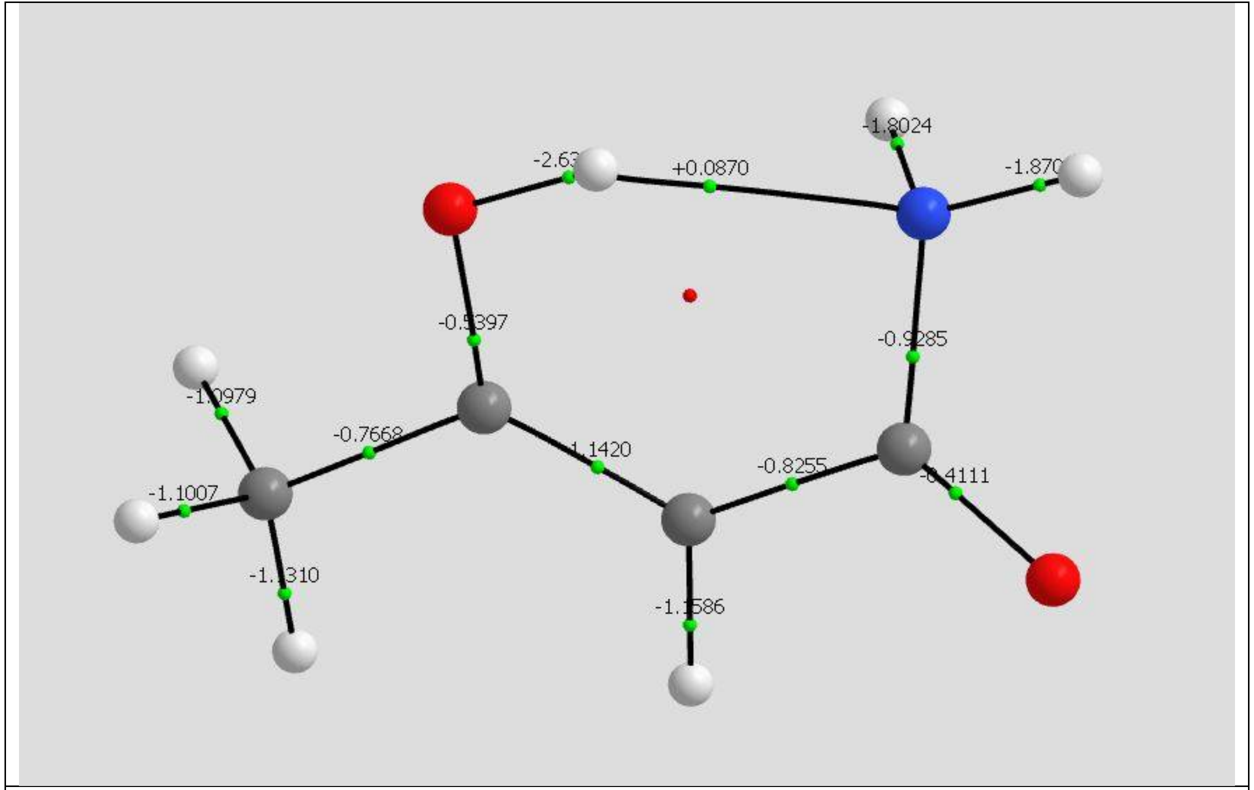

FIVE: Laplacian of the density at Bond Critical Points, including the OH to N H bond

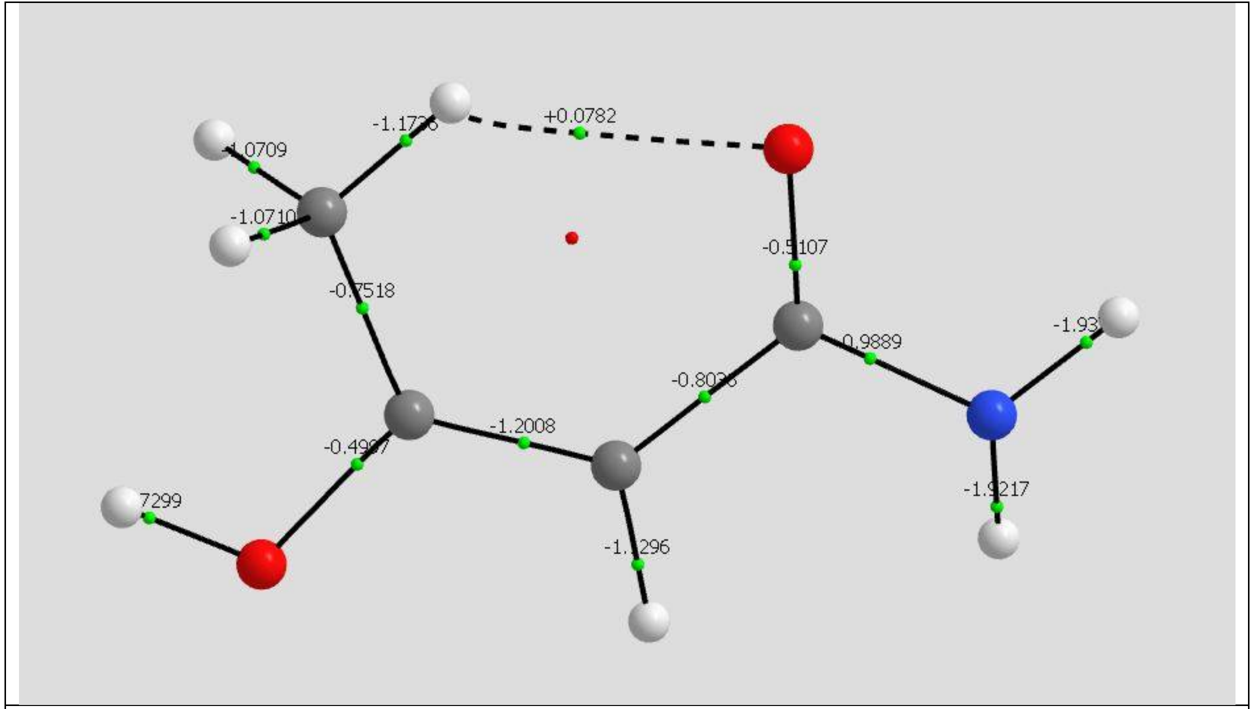

SIX: Laplacian of the density at Bond Critical Points, including the CH to carbonyl O H bond

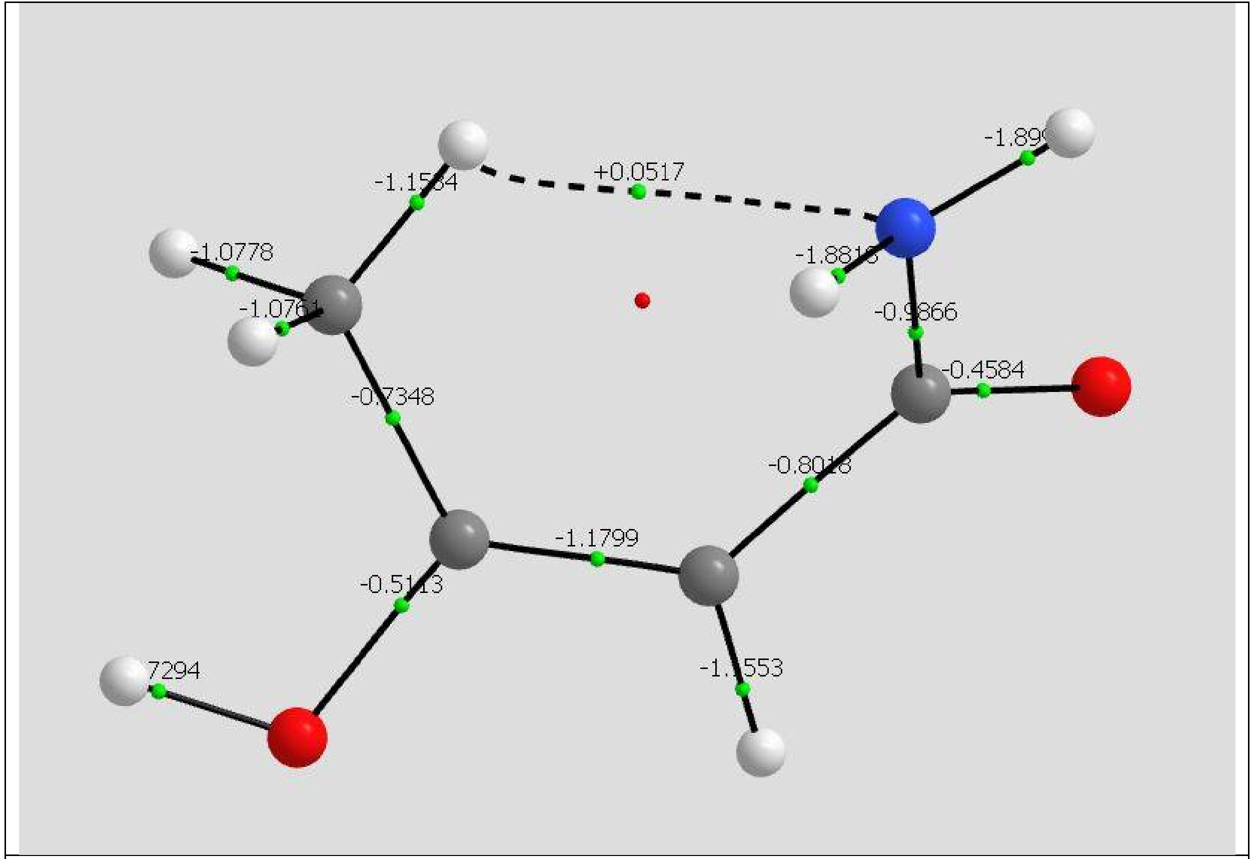

SEVEN: Laplacian of the density at Bond Critical Points, including the CH to N H bond

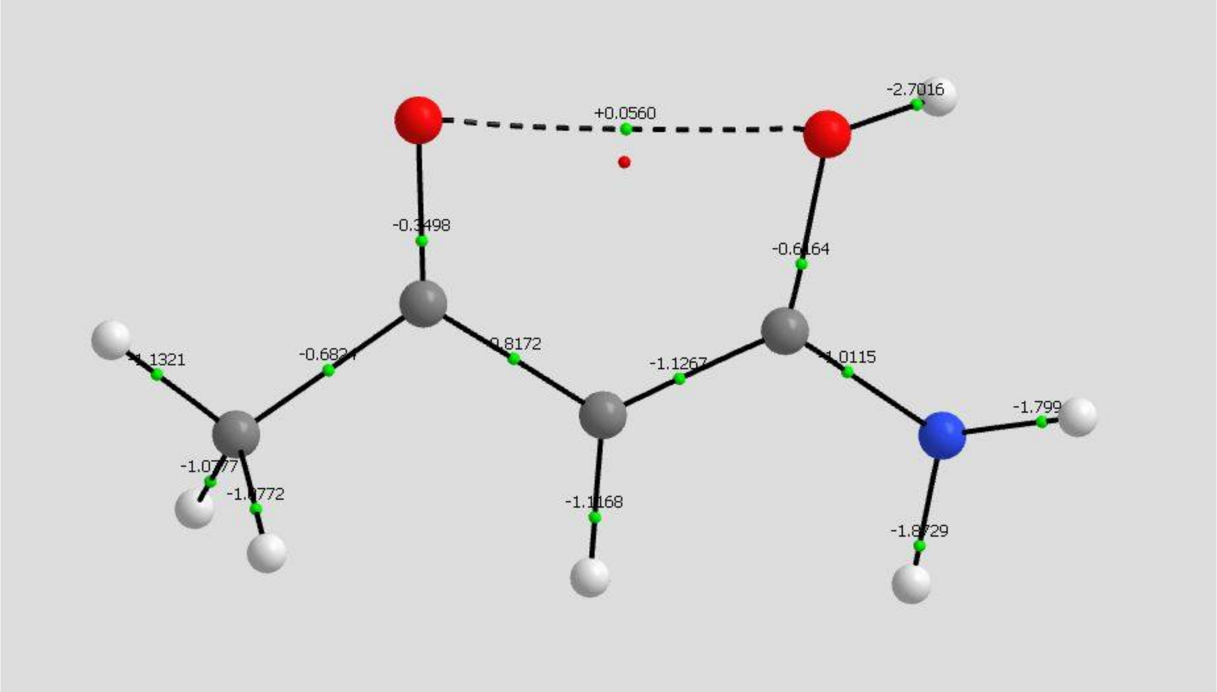

EIGHT: NCI repulsion. Note close approach of RCP and BCP

Figure 2: Topology for species FIVE, SIX, SEVEN, and EIGHT

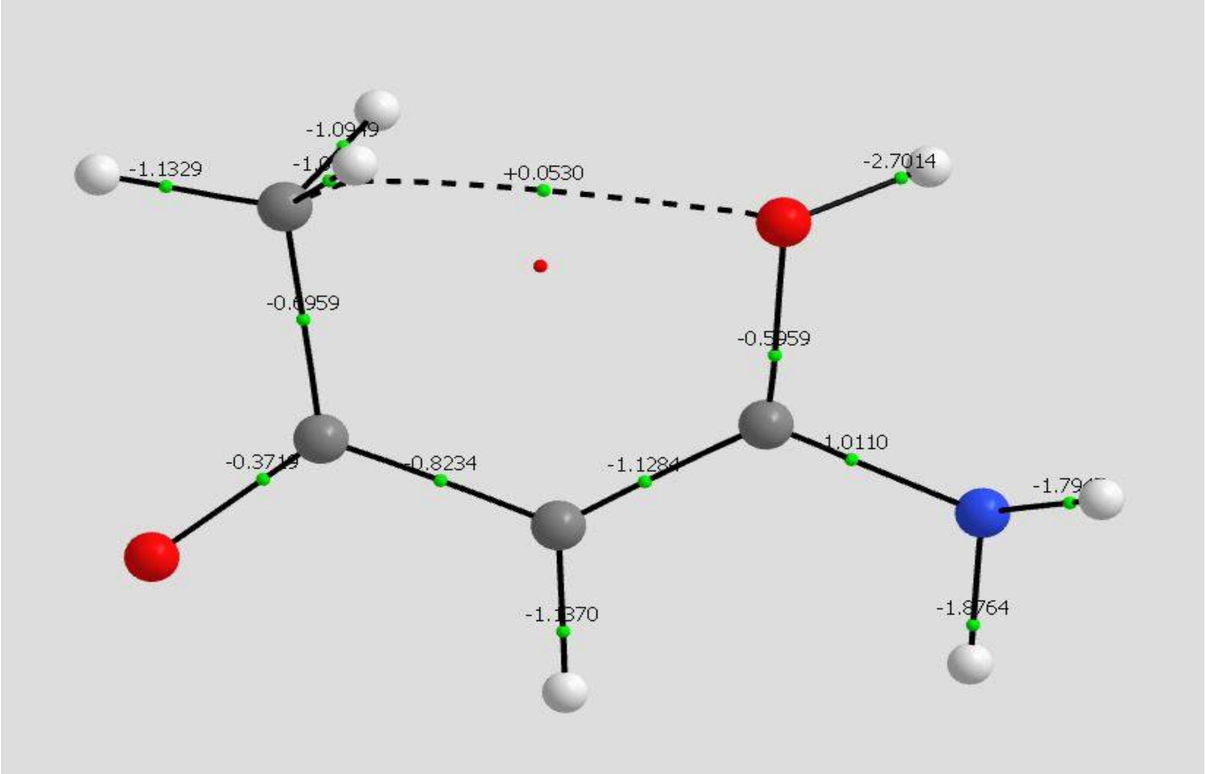

NINE: Laplacian of the density at Bond Critical Points, including the CH…OH H bond

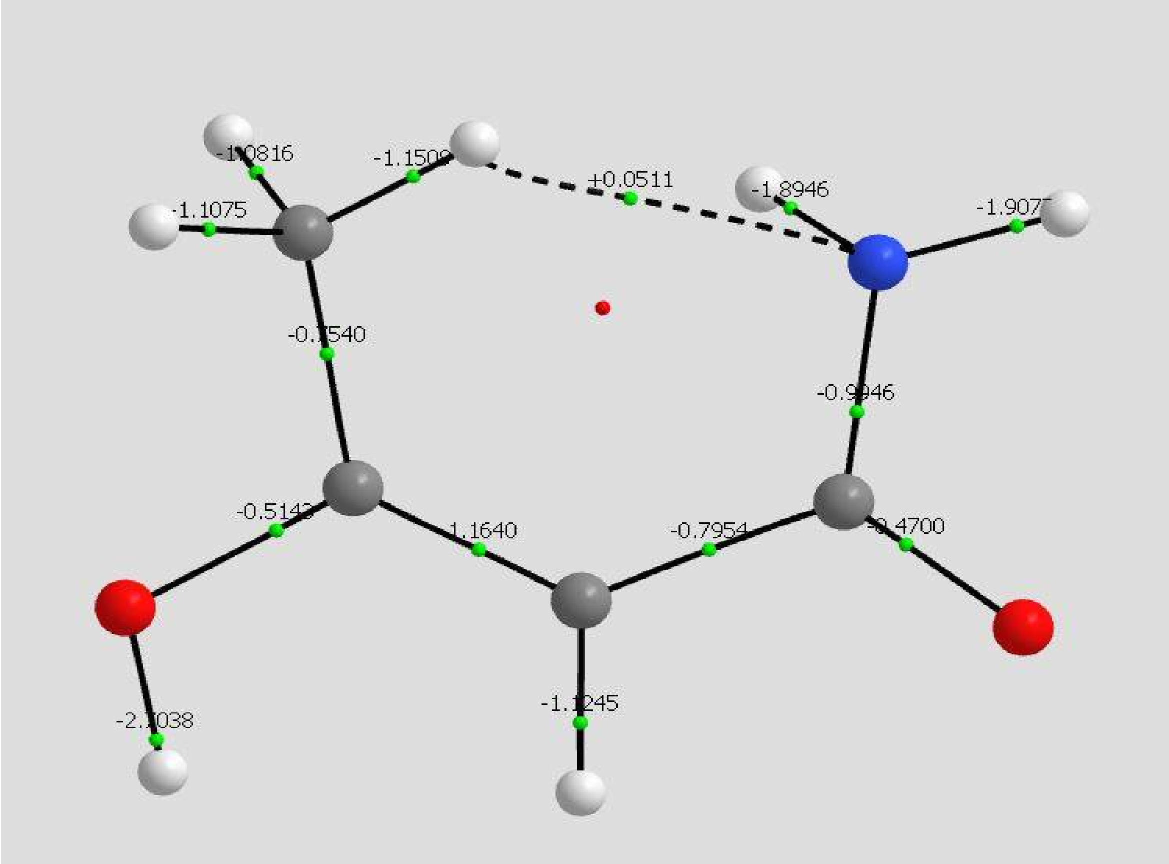

TEN: Laplacian of the density at Bond Critical Points, including the CH to N H bond

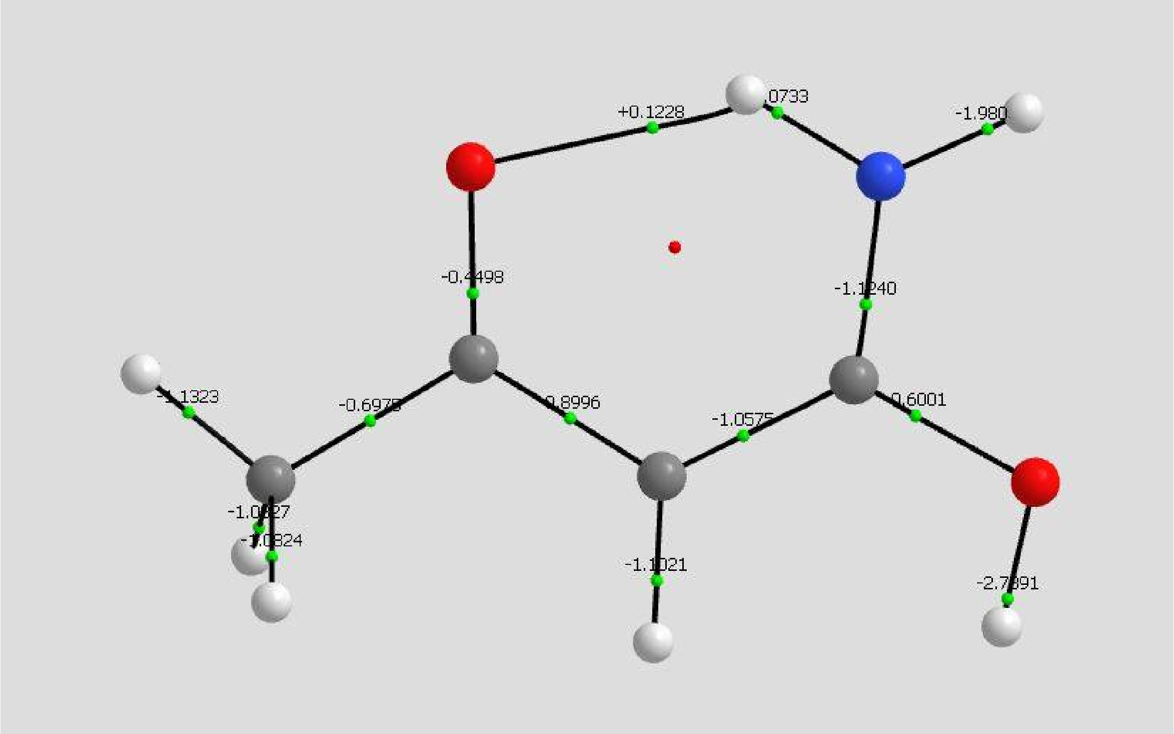

ELEVEN: Laplacian of the density at Bond Critical Points, including the NH to carbonyl O H bond

Figure 3: Species NINE, TEN, and ELEVEN
